# PHD Filter for Object Tracking in Road Traffic Applications Considering Varying Detectability

**DOI:** 10.3390/s21020472

**Published:** 2021-01-11

**Authors:** Olivér Törő, Tamás Bécsi, Péter Gáspár

**Affiliations:** 1 Department of Control for Transportation and Vehicle Systems, Budapest University of Technology and Economics, H-1111 Budapest, Hungary; toro.oliver@mail.bme.hu; 2 Systems and Control Lab, Institute for Computer Science and Control, H-1111 Budapest, Hungary; gaspar.peter@sztaki.mta.hu

**Keywords:** PHD filter, multi-target tracking, detection probability, particle filter, occlusion

## Abstract

This paper considers the object detection and tracking problem in a road traffic situation from a traffic participant’s perspective. The information source is an automotive radar which is attached to the ego vehicle. The scenario characteristics are varying object visibility due to occlusion and multiple detections of a vehicle during a scanning interval. The goal is to maintain and report the state of undetected though possibly present objects. The proposed algorithm is based on the multi-object Probability Hypothesis Density filter. Because the PHD filter has no memory, the estimate of the number of objects present can change abruptly due to erroneous detections. To reduce this effect, we model the occlusion of the object to calculate the state-dependent detection probability. Thus, the filter can maintain unnoticed but probably valid hypotheses for a more extended period. We use the sequential Monte Carlo method with clustering for implementing the filter. We distinguish between detected, undetected, and hidden particles within our framework, whose purpose is to track hidden but likely present objects. The performance of the algorithm is demonstrated using highway radar measurements.

## 1. Introduction

Object detection and tracking in road traffic is a developing field of the automotive industry. Estimation in road traffic environments has several peculiarities. The number of objects to be detected and tracked is usually varying in time, as well as the number of incoming measurements, and possibly the number of sensors mapping the environment is not constant. Objects may enter and leave the observed area, and the visibility in the sensor field of view can change abruptly. The number of available sensors and the possible combinations of them offers a great variety in terms of cost and performance [1,2]. Cost-effectiveness is a major driving force in both development and production. A considerable part of the development and testing of autonomous functions are done in simulated environments [3,4]. For vehicular applications, it is an essential requirement to run an algorithm in real time.

Estimation in road traffic is typically a multi-object problem. Besides the well-established approaches such as the joint probabilistic data association filter (JPDAF) and the multiple hypothesis tracking (MHT), random finite set (RFS)-based solutions started to appear in the last two decades [5]. The multi-object tracking problem covers the estimation of the number of objects present at the scene and the corresponding states. The JPDAF needs to know the number of objects to be tracked and expects the object tracks to be initialized. The MHT works with the assumption that the objects to be detected are point-like, and each generates zero or one measurement in a detector. RFS-based methods are emerging approaches to the multi-object tracking problem. RFS can model object birth and death as well as the clutter process and miss-detection. RFSs can be inserted into the Bayesian framework and used to give recursive estimates about multi-object states. The exact solution to the RFS-based multi-object filtering problem has combinatorial complexity and is generally intractable, thus its solution needs approximations. The probability hypothesis density (PHD) filter [6], which scales linearly with both the number of tracked objects and the measurements, is a practical solution to the multi-object filtering problem that uses first-order approximations. It can be realized with Gaussian mixture [7] or Sequential Monte Carlo [8] approaches.

Automotive applications of the PHD filter have already appeared. Maehlisch et al. used an extended Kalman filter to estimate the ego vehicle state and then used it as a control input in the particle PHD filter to estimate the state of observed traffic participants using LiDAR and camera measurements [9]. Kalyan et al. extracted blobs from 3D LiDAR data and used the Gaussian Mixture PHD filter to track pedestrians in an urban environment [10]. Occlusion of traffic participants poses difficulties in estimation [11]. In the case of partial occlusion, an object could be visible to a sensor; however, the measured intensity or extracted features may be affected [12].

Lin et al. presented a track labeling particle PHD filter wherein birth particles are generated efficiently by assigning more weight to particles close to existing tracks [13].

### 1.1. Related Work

An automotive application of the PHD filter is presented in [14] where vehicles are tracked using features extracted from a monocular camera. Chen et al. reported an underwater tracking application of the PHD and CPHD filters wherein the state-dependent probability of detection values is calculated from a sonar model [15].

An approach to extended target tracking is to model the objects by ellipsoids, which can be represented by positive definite matrices. A multinormal random variable and its covariance matrix from an inverse-Wishart distribution can be inserted into the recursive Bayesian framework to estimate extended target states [16]. The PHD filter using Gaussian inverse-Wishart distributions is reported in [17], and two methods are proposed to partition the measurement set for efficient likelihood computation.

Huang et al. [18] uses the Gaussian inverse-Wishart distribution in the PHD filter to track extended targets and considers the problem to determine whether the source of a measurement is an actual object or clutter.

Zheng and Gao proposed a vehicular application of the PHD filter where roadmap information is used to integrate constraints into the state estimation and fine-tune the process noise covariance matrix to direct the noise along the road. The constraint that the vehicle orientation must be the same as the direction of the road and the velocity vector points in the same direction can be formulated as a linear system of equations and incorporated into the filter [19].

The PHD filter equations have been adopted to the Poisson extended-targets model in [20] to be able to handle extended targets. The applied sensor model involves the combinatorial partitioning of the measurement set, which renders the likelihood computation demanding. Tang et al. derived a multiple detection PHD filter that can be transformed into an extended target or a multisensor PHD filter [21]. Predefined modes represent different measurement models, where the probability of detection is different in each mode. Implementing the multiple detection GM-PHD filter has greater computational requirements, at least a magnitude higher than the classical GM-PHD filter.

The PHD filter’s performance can degrade when a previously observed or tracked target is not detected for a short period [22,23]. In such an event, the PHD mass corresponding to an undetected target is shifted to the detected targets. This phenomenon is addressed as the “spooky effect” at a distance [24]. An approach to reduce this effect is to give a realistic model of the object visibility and consider that the probability of detection is state-dependent, i.e., $p_{d}=p_{d}\left(x\right)$.

Hendeby and Karlsson presented a GM-PHD filter with variable probability of detection. The state-space is divided into segments with different constant $p_{d}$ values. The proposed method approximates the state-dependent probability of detection with a constant value for every Gaussian component [25].

Yazdian-Dehkordi and Azimifar proposed a GM-PHD filter that adopts state-dependent probability of survival. To track and report undetected targets, each Gaussian component is assigned a probability of confirming on which track management is based [26]. Wang et al. presented a SMC-PHD filter that is suitable for scenarios with low probability of detection. The proposed method uses a state-dependent probability of survival to model that objects can enter and exit the sensor field of view. Upon missed detection, the posterior particle weights are revised. False detection and real targets are distinguished with the help of the sequential probability ratio test [27]. Gao et al. proposed a multi-frame GM-PHD filter to manage the weights of Gaussian components corresponding to undetected targets. This approach tries to add inertia to the weights to achieve more reliable track estimates [28].

### 1.2. Contributions of the Paper

In this paper, we address the following issues. As the PHD filter has no memory, its estimation about the number of objects present can change abruptly due to misdetections. To reduce this effect, we model object occlusion to compute a state-dependent probability of detection. This way, the filter can maintain undetected but probably valid hypotheses for a more extended period. Regarding filter realization, we use an SMC method with clusterization based on the work presented in [29]. In our framework, we distinguish detected, undetected, and hidden particles. The purpose of hidden particles is to help the tracking of undetected but probably present objects. This approach enables the creation of labeled track estimates without augmenting the state vector with a label variable. The performance of the proposed filter is analyzed in challenging simulations.

The paper is organized as follows. In Section 2, we begin by summarizing the theoretical background and providing motivation for the presented work. Section 3 details the proposed filter. The occlusion model for computing a state-dependent probability of detection is detailed in Section 4. In Section 5 we evaluate the filter performance in abstract simulations and simple traffic scenarios. Section 6 presents a case study with highway radar measurement to validate the proposed method. The results are discussed in Section 7.

## 2. Theoretical Background

This section summarizes the theoretical concepts needed for the proposed method. After presenting the original PHD filter, we discuss particle filter implementation questions focusing on road traffic applications. In a multi-target scenario, the number of objects to be tracked is, in general, unknown a priori and time-dependent. Similarly, the set of incoming measurements has variable cardinality. Since the PHD filter equations are defined over the single-object state-space, no multi-object motion and measurement models are needed.

The discrete dynamic model that will be used throughout this work consists of the equations

(1) $$\begin{array}{cc} x_{k+1} & =f_{k}\left(x_{k}\right)+w_{k} \end{array}$$

(2) $$\begin{array}{cc} z_{k} & =h_{k}\left(x_{k}\right)+v_{k}\;, \end{array}$$

where $f_{k}$ is the state transition function, $h_{k}$ is the measurement function and *k* stands for the time index. The uncertainties are modeled as additive zero mean white Gaussian noises with covariance matrices Cov[w]=Q and Cov[v]=R.

### 2.1. Random Finite Sets

A random finite set *X* consisting of random vector variables takes the form $X=\{x_{1},\ldots ,x_{n}\}$, where the cardinality |X|=n is also a random number, distributed according to ϱ(n). The elements in the random set are unordered, making every permutation equivalent. The mathematical apparatus providing methods to carry out calculations with RFSs is called finite set statistics (FISST), summarized in the monograph [30]. The FISST PDF can describe the distribution of an RFS:

(3) $$f\left(X\right)=n!\;\varrho \left(n\right)\;p_{n}\left(x_{1},\ldots ,x_{n}\right)\;,$$

where $p_{n}\left(x_{1},\ldots ,x_{n}\right)$ is the symmetric joint distribution of random vectors $x_{1},\ldots ,x_{n}$ and ϱ(n) is the cardinality distribution. A Bayes type multi-object filter can be formulated with RFSs; however, the exact solution is generally not tractable due to its combinatorial complexity. A computationally tractable solution to the recursive Bayes multi-object filtering problem can be derived in numerous ways [31]. An approach that leads to a linearly scaling filter is to approximate the full multi-object density f(X) with its first-order moment resulting in the probability hypothesis density (PHD) filter.

The first-order moment of the FISST PDF f(X) defined over the single-object state-space is written as

(4) $$D\left(x\right)=\int \delta_{X}\left(x\right)f\left(X\right)\;\delta X\;\;.$$

The scalar-valued PHD or intensity function defined in (4) takes higher values at locations in the state-space where an object is likely to be found. Integrating D(x) over a region Ω in the state-space yields the expectation of the cardinality of *X*, which is the expected number of objects in that region:

(5) $$\int_{\Omega }\;D\left(x\right)dx=E\left[|X|\right]\;.$$

### 2.2. PHD Filter

The PHD filter is a recursive algorithm formulated in the Bayes framework. However, instead of the full multi-object PDF, only the PHD function is propagated through the recursion. The PHD filter, to achieve closed-form formulas, uses the Poisson RFS to model targets. The FISST density function of a Poisson RFS is

(6) $$f\left(X\right)=e^{-\lambda }\prod_{x\in X}\lambda p\left(x\right)\;,$$

and the distribution of the cardinality is Poisson with parameter λ:

(7) $$\varrho \left(n\right)=\frac{e^{-\lambda }\lambda^{n}}{n!}\;.$$

The PHD function of a Poisson RFS is

(8) D(x)=λp(x),

which integrates to λ.

Given the PHD at timestep k−1 as $D_{k-1|k-1}\left(x\right)$ the prediction equation of the PHD filter is

(9) $$D_{k|k-1}\left(x\right)=\gamma_{k|k-1}\left(x\right)+p_{s}\int \pi_{k|k-1}\left(x|x^{\prime }\right)D_{k-1|k-1}\left(x^{\prime }\right)dx^{\prime }\;,$$

where $\pi_{k|k-1}\left(x|x^{\prime }\right)$ stands for the transition density from timestep k−1 to *k* and $p_{s}$ is the probability of object survival. Objects appearing between timestep k−1 and *k* are represented by the birth PHD $\gamma_{k|k-1}\left(x\right)$. Updating the PHD is carried out according to

(10) $$D_{k|k}\left(x\right)=\left[1-p_{d}\left(x\right)+\sum_{z\in Z_{k}}\frac{p_{d}\left(x\right)g_{k}\left(z|x\right)}{\kappa \left(z\right)+\int p_{d}\left(x\right)g_{k}\left(z|x\right)D_{k|k-1}\left(x\right)\;dx}\right]D_{k|k-1}\left(x\right)\;,$$

where $p_{d}\left(x\right)$ is the probability of detection at location x in the state-space, $g_{k}\left(z|x\right)$ is the measurement likelihood function and κ(z) represents the clutter density from where false measurements are originating.

It is beneficial to construct the birth PHD $\gamma_{k|k-1}\left(x\right)$ with the help of the measurements $z_{k-1}$ [29]. This way, new objects will only appear in regions of the state-space that are covered by measurements. The birth PHD is computed via the time-prediction integral as

(11) $$\gamma_{k|k-1}\left(x\right)=p_{s}\int \pi_{k|k-1}\left(x|x^{\prime }\right)\gamma_{k-1}\left(x^{\prime }\right)dx^{\prime }\;,$$

where $\gamma_{k-1}\left(x\right)$ is the spatial PDF of object birth assembled as a mixture:

(12) $$\gamma_{k-1}\left(x|Z_{k-1}\right)=\frac{1}{|Z_{k-1}|}\sum_{z\in Z_{k-1}}\beta \left(x|z\right)\;.$$

The density β(x|z) represents the image of a measurement in the state-space.

Regarding filter implementation, practical methods are the Gaussian mixture and particle filter approaches. In this work, we concentrate on the particle filter implementation, which is based on the weighted sum approximation of the PHD function using *N* samples at locations $x^{\left(i\right)}$ with weights $w^{\left(i\right)}$:

(13) $$D\left(x\right)\approx \sum_{i=1}^{N}w^{\left(i\right)}\;\delta_{x^{\left(i\right)}}\left(x\right)\;.$$

### 2.3. Problem Statement and Motivation

The classical PHD filter uses the standard measurement model that makes the assumptions that a target can generate at most one measurement, and a measurement cannot be generated by more than one target [20]. In this case, the PHD filter has linear scaling properties in terms of the tracked objects and in the number of processed measurements.

Implementing the PHD filter with particle approximation can be done relatively easily, as described in [30] (Chapter 16.5.2). The PHD filter does not produce discrete object state estimates. It must be extracted from the PHD function, either represented by an analytical formula or a particle system. Algorithms such as k-means or expectation–maximization can be used to create particle clusters. The number of clusters to be created is guided by the expected number of objects, which is the sum of particle weights. This expectation is, however, not stable and requires averaging [30] (p. 623).

Updating the PHD function using particle approximation can be done, according to (10), in one step. This standard approach, referred to as the pseudo-likelihood update, has drawbacks making the filtering less effective [32]. On the one hand, if measurements do not support a particle, its weight will be decreased by the factor $1-p_{d}$. If an object is absent from the measurement set due to miss-detection, but present at the scene, the filter is unlikely to resample the corresponding particles. Consequently, upon re-detecting the object, particles need to be drawn again from the birth pool. On the other hand, extracting the posterior multi-object state from the particles is not straightforward. The clustering algorithm needed to give object state estimates increases the computational requirement and is essentially ad-hoc.

In case an object is temporarily occluded, particles representing it are likely to be dropped by the filter in a few timesteps, and no point estimate could be created. If the occlusion is anticipated by means of adjusting the probability of detection to a low value, undetected particles can exist for a longer time. This can be illustrated by writing (10) as a particle approximation:

(14) $$w_{k|k-1}^{\left(i\right)}=\left[1-p_{d}\left(x_{k|k-1}^{\left(i\right)}\right)\right]w_{k|k-1}^{\left(i\right)}+\left[\sum_{z\in Z_{k}}\frac{p_{d}\left(x_{k|k-1}^{\left(i\right)}\right)g_{k}\left(z|x_{k|k-1}^{\left(i\right)}\right)}{\kappa \left(z\right)+\sum_{j=1}^{N}p_{d}\left(x_{k|k-1}^{\left(j\right)}\right)g_{k}\left(z|x_{k|k-1}^{\left(j\right)}\right)w_{k|k-1}^{\left(j\right)}}\right]w_{k|k-1}^{\left(i\right)}\;,$$

where the first term on the right-hand side stands for the undetected, the second term for the detected particle weights. At positions in the state-space where $p_{d}\left(x\right)$ has low values, undetected particle weights will reduce by a small amount helping particles to exist longer.

Erdinc et al. analyzed the effect of missed detection to the expected number of objects in a single-target scenario with constant $p_{d}$ [22]. The underestimation of the expected target number is due to the first-order approximation used in the PHD filter [33] (p. 199). If no measurements arrive at timestep *k* then the PHD filter estimates

(15) $$N_{k|k}=\left(1-p_{d}\right)N_{k|k-1}\;,$$

while the exact formula would be

(16) $$N_{k|k}=\frac{\left(1-p_{d}\right)N_{k|k-1}}{1-N_{k|k-1}p_{d}}\;.$$

The difference is negligible if $p_{d}$ is close to unity but significant otherwise.

We mention one more aspect that is relevant to the current application. Since the birth density is generated from the measurements, multiple detections of the same object must be considered. An individual object may be detected numerous times either because it is extended, or the sensor’s refresh rate is higher than the filtering timestep. In this case, the birth density would gain more weight, which can be desired or undesired. On the other hand, the expected number of newborn or already present objects is biased, which is clearly undesired.

## 3. The Proposed Method

Our approach builds on the PHD particle filter algorithm described in [29], wherein the central idea is to create particle clusters in a probabilistic manner, using the measurement set. Each particle cluster is updated independently by a standard bootstrap particle method. A discrete object is extracted from a cluster and reported by the filter if enough particle weight is accumulated in one. The number of particle clusters and the number of reported objects are upper bounded by the measurement set cardinality. The clustering method above uses a sensor model that generates zero or one measurement per object. This assumption ensures that a probability distribution can be generated that measures how likely a particle belongs to a measurement. It should be noted that only targets that have been detected at the current timestep have a chance to be reported.

We aim to track and report objects that are undetected but possibly present. To achieve this, the proposed filter distinguishes detected, hidden, and undetected particles. The difference between undetected and hidden particles is that objects can be reported based on hidden particles if enough particle weight is concentrated. Suppose the filter considers a cluster of particles to be an object that should be reported based on the sum of the contained particle weights. In that case, the point estimate and the object state’s covariance is stored with the associated label. In the next iteration, birth particles are generated using measurements from the previous timestep. Every particle and the stored point estimates are propagated according to the motion model. In the update step, we create the union of the collected measurements and the stored point estimates and with a given threshold merge the close matches.

The update method for hidden particles is the same as for undetected particles; however, the same clusterization method is used as for detected particles. The likelihood computation happens with the augmented measurement set. For the real measurements, the covariance matrix *R* is used while for the point estimate components the covariance matrix computed from the sample is substituted into the likelihood function. The resultant likelihood values are used to create particle clusters but not to update particle weights. The actual update step happens cluster-wise with the associated measurement or object generated pseudo-measurement. Hidden particles are updated as undetected particles. This way we introduce no additional information by increasing particle weights with the pseudo-measurements.

The filter is initialized with the assumption that there are no detected objects, thus with zero particle. Objects will emerge from the measurement generated birth particles. For every measurement $n_{p}$ particles are drawn from the birth pool, which is defined by the inverse measurement model $h^{-1}\left(z\right)$. Besides the measurement generated birth particles, the filter uses birth particles originating from the assumed hidden objects. A hidden object is a reported point estimate computed from a hidden particle cluster.

At timestep k−1 let ${\left\{x_{k-1}^{\left(i\right)},w_{k-1}^{\left(i\right)}\right\}}_{i=1}^{N_{k-1}}$ denote the particle system approximating the posterior PHD function $D_{k-1}\left(x\right)$. Beside particles, reported object states and labels are also propagated in the recursion. Labels are discrete identifiers, typically a unique integer number for every object. Let $O_{k-1}$ denote the set of estimated object states, covariance matrices and labels with cardinality $|O_{k-1}|=o_{k-1}$ thus

(17) $$O_{k-1}={\left\{c_{k-1}^{\left(i\right)},C_{k-1}^{\left(i\right)},l_{k-1}^{\left(i\right)}\right\}}_{i=1}^{o_{k-1}}\;,$$

where $c_{k-1}$ is the estimated state and $C_{k-1}$ is the error covariance matrix and $l_{k-1}$ is the label. Similarly, the set of hidden objects is

(18) $$O_{k-1,h}={\left\{c_{k-1,h}^{\left(i\right)},C_{k-1,h}^{\left(i\right)},l_{k-1}^{\left(i\right)}\right\}}_{i=1}^{o_{k-1,h}}\;,$$

where $|O_{k-1}|=o_{k-1,h}$. The measurement set from timestep *k* is $Z_{k}=\left\{z_{k}^{\left(1\right)},\ldots ,z_{k}^{\left(m_{k}\right)}\right\}$.

For birth particles the distribution to be drawn from is created as a mixture generated by the measurements and the hidden object estimates. For the measurement driven birth pool the procedure is the following [34] (p. 68). Given $m_{k-1}$ measurements the birth pool $b_{k-1,z}\left(x|Z_{k-1}\right)$ is created by inverting h(x) for every measurement yielding the mixture

(19) $$b_{k-1,z}\left(x|Z_{k-1}\right)=\sum_{i=1}^{m_{k-1}}\beta_{k-1}\left(x|z_{k-1}^{\left(i\right)}\right)\;,$$

where the individual Gaussians have the form

(20) $$\beta_{k-1}\left(x|z_{k-1}^{\left(i\right)}\right)=N\left(x;h_{k-1}^{-1}\left(z_{k-1}^{\left(i\right)}\right),H_{k-1}R_{k-1}H_{k-1}^{⊤}\right)\;.$$

For the birth particles that are generated by the hidden objects, the birth pool is

(21) $$b_{k-1,h}\left(x|O_{k-1,h}\right)=\sum_{i=1}^{o_{k-1,h}}\beta_{k-1,h}\left(x|c_{k-1,h}^{\left(i\right)}\right)\;,$$

where the individual Gaussians are

(22) $$\beta_{k-1,h}\left(x|c_{k-1,h}^{\left(i\right)}\right)=N\left(x;c_{k-1,h}^{\left(i\right)},C_{k-1,h}^{\left(i\right)}\right)\;.$$

The overall birth density function assembles as a normalized mixture:

(23) $$b_{k-1}\left(x|Z_{k}⋃O_{k-1,h}\right)=\frac{1}{m_{k-1}}b_{k-1,z}\left(x|Z_{k-1}\right)+\frac{1}{o_{k-1,h}}b_{k-1,h}\left(x|O_{k-1,h}\right)\;.$$

Sampling from the distributions $b_{k-1}$ results the birth particle system $\left\{x_{b,k-1}^{\left(i\right)},w_{b,k-1}^{\left(i\right)}\right\}$${}_{i=1}^{N_{b,k-1}}$ where the weights are set such that the particles represent $\nu_{b,k-1}$ objects: $w_{b,k-1}^{\left(i\right)}=\nu_{b,k-1}/N_{b,k-1}$ and the total number of birth particles is

(24) $$N_{b,k-1}=n_{p}\left(m_{k-1}+o_{k-1,h}\right)\;.$$

The posterior birth intensity, $\nu_{b,k}$ can be evaluated, according to [35], by summing up the newborn particle weights and if it is found to be unrealistic the prior $\nu_{b,k-1}$ values can be modified, and changes propagated to the update step.

The particles coming from timestep k−1 and the birth particles create the ensemble ${\left\{x_{k-1}^{\left(i\right)},w_{k-1}^{\left(i\right)}\right\}}_{i=1}^{N_{k-1}+N_{b,k-1}}$ on which the prediction step is performed according to the motion model:

(25) $$x_{k|k-1}^{\left(i\right)}∼N\left(f_{k-1}\left(x_{k-1}^{\left(i\right)}\right),Q_{k-1}\right)\;,$$

and the predicted weights are

(26) $$w_{k|k-1}^{\left(i\right)}=p_{s}w_{k-1}^{\left(i\right)}\;.$$

Beside particles, the object states and covariances are also propagated as

(27) $$\begin{array}{cc} c_{k|k-1}^{\left(i\right)} & =f_{k-1}\left(c_{k-1}^{\left(i\right)}\right) \end{array}$$

(28) $$\begin{array}{cc} C_{k|k-1}^{\left(i\right)} & =F_{k-1}^{\left(i\right)}C_{k-1}^{\left(i\right)}F_{k-1}^{(i)⊤}+Q_{k-1}\;\left(i=1\ldots o_{k-1}\right) \end{array}$$

and

(29) $$\begin{array}{cc} c_{k|k-1,h}^{\left(j\right)} & =f_{k-1}\left(c_{k-1,h}^{\left(j\right)}\right) \end{array}$$

(30) $$\begin{array}{cc} C_{k|k-1,h}^{\left(j\right)} & =F_{k-1}^{\left(j\right)}C_{k-1,h}^{\left(j\right)}F_{k-1}^{(j)⊤}+Q_{k-1}\;\left(j=O_{k-1}+1\ldots o_{k-1,h}\right)\;, \end{array}$$

where $F_{k-1}^{\left(i\right)}$ is the Jacobian of $f_{k-1}$ evaluated at $c_{k-1}^{\left(i\right)}$ or $c_{k-1,h}^{\left(i\right)}$ and the labels are propagated without change: $l_{k|k-1}^{\left(i\right)}=l_{k-1}^{\left(i\right)}$.

At the update step, likelihood values for every particle-measurement pair must be computed. At this stage, particles will be classified as detected, hidden, or not detected. The predicted but not detected objects will be considered to be pseudo-measurements taking values from (27)–(30).

The algorithm to match the predicted objects and measurements considers only the position coordinates, thus works on a two-dimensional marginal distribution. The Kullback–Leibler (KL) divergence is used to compare measurements and the predicted objects. For every measurement-object pair, a KL divergence value is computed. The null hypothesis that the measurement does not belong to any object is represented by a uniform distribution covering the area of interest. The size of the area sets the intensity of the distribution.

The KL divergence between two *n*-dimensional normal distribution with means $\mu_{1},\mu_{2}$ and covariance matrices $\Sigma_{1},\Sigma_{2}$ is

(31) $$D_{KL}\left(N_{1},N_{2}\right)=\frac{1}{2}\left(\log\frac{\det\Sigma_{2}}{\det\Sigma_{1}}-n+\mathrm{tr}\left(\Sigma_{2}^{-1}\Sigma_{1}\right)+{\left(\mu_{2}-\mu_{1}\right)}^{⊤}\Sigma_{2}^{-1}\left(\mu_{2}-\mu_{1}\right)\right)\;.$$

For the null hypothesis, the KL divergence between a uniform U(Γ) and a normal distribution is computed:

(32) $$D_{KL}\left(U(\Gamma ),N(\mu ,\Sigma )\right)=\int_{\Gamma }U\left(\Gamma \right)\log\frac{U(\Gamma )}{N(\mu ,\Sigma )}dx\;.$$

Considering a rectangular region of Γ=(b−a)(d−c) and a two-dimensional normal distribution the integral evaluates to

(33) $$\begin{array}{c} D_{KL}\left(U(\Gamma ),N(\mu ,\Sigma )\right)=\log\frac{2\pi \sqrt{\det\Sigma }}{\Gamma }+\\ +\frac{1}{6\Gamma }\left(\sigma_{11}\left(a^{3}-b^{3}\right)\left(c-d\right)+\frac{3}{2}\sigma_{12}\left(a^{2}-b^{2}\right)\left(c^{2}-d^{2}\right)+\sigma_{22}\left(c^{3}-d^{3}\right)\left(a-b\right)\right) \end{array}$$

where $\sigma_{ij}$ are the elements of Σ.

An object, be it hidden or detected, is considered to be covered by a measurement *j* if

(34) $$D_{KL}\left(N\left(z_{k}^{\left(j\right)},R\right),N\left(c,C\right)\right)>D_{KL}\left(U\left(\Gamma_{k}\right),N\left(c,C\right)\right)$$

in which case the object c, which can be $c_{k|k-1}^{\left(i\right)}$ or $c_{k|k-1,h}^{\left(i\right)}$ is appended to the measurement set.

Depending on whether the measurements come with labels, two strategies can be used. In the case of labeled measurements, the received label is used if no object can be associated. If an object matches the measurement, then the received label will be replaced with the object’s label. In unlabeled measurements, an unmatched measurement should be assigned an unused label chosen from a designed pool.

The augmented set ${\hat{Z}}_{k}⊇Z_{k}$ may or may not contain more elements than the original measurement set and $|{\hat{Z}}_{k}\left|=\right|Z_{k}|+{\hat{o}}_{k}+{\hat{o}}_{k,h}$ where ${\hat{o}}_{k}$ and ${\hat{o}}_{k,h}$ are the number of detected and hidden object inserted into the set. The numbers ${\hat{o}}_{k}+{\hat{o}}_{k,h}$ are coming from the condition (34): if the inequality holds for a certain object then $o_{k}$ or $o_{k,h}$ is incremented, depending on whether it was a detected or hidden object. Using ${\hat{Z}}_{k}$ the particles are partitioned to form clusters. Partitioning is performed in a probabilistic manner as described in [32]. A probability is computed to every particle-measurement pair $\left(x_{k|k-1}^{\left(i\right)},{\hat{z}}_{k}^{\left(j\right)}\right),\;\;{\hat{z}}_{k}^{\left(j\right)}\in {\hat{Z}}_{k}$ which measures how likely measurement ${\hat{z}}_{k}^{\left(j\right)}$ is generated by an object at state $x_{k|k-1}^{\left(i\right)}$:

(35) $$P_{ij}=\frac{p_{d}\left(x_{k|k-1}^{\left(i\right)}\right)g_{k}\left({\hat{z}}_{k}^{\left(j\right)}|x_{k|k-1}^{\left(i\right)}\right)w_{k|k-1}^{\left(i\right)}}{\kappa \left(z_{j}\right)+\sum_{l=1}^{N_{k|k-1}}p_{d}\left(x_{k|k-1}^{\left(l\right)}\right)g_{k}\left({\hat{z}}_{k}^{\left(j\right)}|x_{k|k-1}^{\left(l\right)}\right)w_{k|k-1}^{\left(l\right)}}\;,$$

where $N_{k|k-1}=N_{k-1}+N_{b,k-1}$. The likelihood value for a measurement is

(36) $$g_{k}\left({\hat{z}}_{k}^{\left(j\right)}|x_{k|k-1}^{\left(i\right)}\right)=N\left({\hat{z}}_{k}^{\left(j\right)};h_{k}\left(x_{k|k-1}^{\left(i\right)}\right),R\right)\;,\;\mathrm{for}\;j=1\ldots \left|Z_{k}\right|$$

and for a pseudo-measurement

(37) $$g_{k}\left({\hat{z}}_{k}^{\left(j\right)}|x_{k|k-1}^{\left(i\right)}\right)=N\left(c;h_{k}\left(x_{k|k-1}^{\left(i\right)}\right),C\right)\;,$$

where c is a detected object for $j=|Z_{k}\left|+1,\ldots ,\right|Z_{k}|+{\hat{o}}_{k}$ and is a hidden object for $j=|Z_{k}|+{\hat{o}}_{k}+1,\ldots ,\left|Z_{k}\right|+{\hat{o}}_{k}+{\hat{o}}_{k,h}$. A particle is regarded as not detected with probability

(38) $$P_{i0}=\left(1-p_{d}\left(x_{k|k-1}^{\left(i\right)}\right)\right)w_{k|k-1}^{\left(i\right)}\;.$$

Normalizing the probabilities $P_{ij},\;(j=0,1,\ldots ,|{\hat{Z}}_{k}\left|\right)$ generates a discrete distribution above the indices *j* for every particle *i*:

(39) $$p_{i}\left(j\right)=\frac{P_{ij}}{\sum_{l=0}^{|{\hat{Z}}_{k}|}P_{il}}\;.$$

For every particle *i* an index *j* is chosen with probability $p_{i}\left(j\right)$ thus generating $1+|{\hat{Z}}_{k}|$ clusters. Particle clusters are categorized according to the value of *j* as:

$$\begin{array}{cc} \mathrm{not}\;\mathrm{detected}: & j=0\\ \mathrm{detected}\;\mathrm{particle}: & 0<j<=|Z_{k}|\\ \mathrm{hidden}\;\mathrm{particle}: & |Z_{k}\left|<j<=\right|{\hat{Z}}_{k}| \end{array}$$

The formed particle clusters are denoted by ${\left\{{𝒞}_{k}^{\left(j\right)},l_{k}^{\left(j\right)}\right\}}_{j=0}^{|{\hat{Z}}_{k}|}$, where $l_{k}^{\left(j\right)}$ is the label associated with the cluster, which is the same as the label of the measurement the particles in the cluster were assigned to. The particle weights in the hidden and the not detected clusters are updated the same way:

(40) $$w_{k|k}^{\left(i\right)}=\left(1-p_{d}\left(x_{k|k-1}^{\left(i\right)}\right)\right)w_{k|k-1}^{\left(i\right)}\;.$$

Particle weights in the detected clusters are computed using a bootstrap particle filter update:

(41) $$w_{k|k}^{\left(i\right)}=\frac{p_{d}\left(x_{k|k-1}^{\left(i\right)}\right)g_{k}\left(z_{k}^{\left(j\right)}|x_{k|k-1}^{\left(i\right)}\right)w_{k|k-1}^{\left(i\right)}}{\kappa \left(z_{j}\right)+\sum_{l=1}^{N_{k|k-1}}p_{d}\left(x_{k|k-1}^{\left(l\right)}\right)g_{k}\left(z_{j}|x_{k|k-1}^{\left(l\right)}\right)w_{k|k-1}^{\left(l\right)}}\;.$$

For the not detected and hidden clusters the updated and predicted particles states are identical:

(42) $$x_{k|k}^{\left(i\right)}=x_{k|k-1}^{\left(i\right)}\;,$$

while for detected clusters, particles are resampled with probability proportional to their weights.

The sum of particle weights in a detected or hidden cluster is between 0 and 1 thus it is regarded as the existence probability of an object represented by the particles:

(43) $$r_{k}^{\left(j\right)}=\sum_{i=1}^{|{𝒞}_{k}^{\left(j\right)}|}w_{k|k}^{\left(i\right)}\;.$$

If $r_{k}^{\left(j\right)}$ is above a given threshold, a point estimate is computed from the particle cluster, and an object is registered as detected or hidden, based on the index *j*. The detected object set is

(44) $$O_{k}={\left\{c_{k}^{\left(i\right)},C_{k}^{\left(i\right)},l^{\left(i\right)}\right\}}_{i=1}^{o_{k}}\;,$$

where the point estimates are computed cluster-wise as a weighted sum of the corresponding particle states:

(45) $$c_{k}^{\left(i\right)}=\sum_{j=1}^{|{𝒞}_{k}^{\left(\alpha \right)}|}w_{k|k}^{\left(j\right)}x_{k|k}^{\left(j\right)}\;$$

for a cluster $|C_{k}^{\left(\alpha \right)}|$, $\alpha \in 1,\ldots ,|Z_{k}|$. The error covariance matrices are computed from the samples:

(46) $$C_{k}^{\left(i\right)}=\sum_{j=1}^{|{𝒞}_{k}^{\left(\alpha \right)}|}w_{k|k}^{\left(i\right)}\left(c_{k}^{\left(i\right)}-x_{k|k}^{\left(j\right)}\right){\left(c_{k}^{\left(i\right)}-x_{k|k}^{\left(j\right)}\right)}^{⊤}\;.$$

For hidden objects, the calculations are the same for indices $\alpha \in |Z_{k}\left|+1,\ldots ,\right|{\hat{Z}}_{k}|$ resulting in the set

(47) $$O_{k,h}={\left\{c_{k,h}^{\left(i\right)},C_{k,h}^{\left(i\right)},l^{\left(i\right)}\right\}}_{i=1}^{o_{k,h}}\;.$$

It should be noted that more sophisticated procedures can be carried out to extract point estimates from particle clouds. Differentiating newborn and already persisting particles and normalizing weight can improve estimation performance, as reported in [36,37].

### Handling Multiple Measurements in One Timestep

Since the PHD filter uses the standard measurement model, it can only perform efficiently if an object cannot generate more than one measurement. In other words, multi-detection does not arise.

Due to the high refresh rate, radar can detect and report an object multiple times during the filtering timestep. To handle the multiple detection instead of using a non-standard measurement model that captures this peculiarity, we process the measurements online. We collect the measurements that arrived during the filter timestep and form unique clusters. We use the fact that vehicles cannot overlap in the position subspace of the state-space. The algorithm is summarized in Algorithm 1.
**Algorithm 1** Measurement clusterization.1:**procedure** CreateUniqueClusters(*Z*)2:    From measurement set $Z={\left\{z_{i}\right\}}_{i=1}^{m}$ create matrix $Z=[z_{1},\ldots ,z_{m}]$3:    Sort the columns of Z in increasing order by the rows 1 and 2Segment Z to sets $C_{n}$ as follows:4:    n=1, $C_{1}=\left\{Z_{*,1}\right\}$                              ▹* denotes all elements5:    **for** i←2 to *m* **do**6:        **if** $\left(Z_{1,i}-Z_{1,i-1}\right)<d_{1}\wedge \left(Z_{2,i}-Z_{2,i-1}\right)<d_{2}$**then**▹ Given thresholds $d_{1}$ and $d_{2}$7:           $C_{n}=C_{n}⋃\left\{Z_{*,i}\right\}$8:        **else**9:           n=n+110:           $C_{n}=\left\{Z_{*,i}\right\}$11:        **end if**12:    **end for**Compute average state vector $c_{i}$ from every set $C_{i}$:13:    **for** i←1 to *n* **do**14:        $c_{i}=\frac{1}{|C_{i}|}\sum_{\alpha \in C_{i}}\alpha$15:    **end for**16:    **return** $Z^{*}=\left\{c_{1},\ldots ,c_{m}\right\}$                             ▹ Clustered measurements17:**end procedure**

## 4. Occlusion Model

The $p_{d}\left(x\right)$ coefficient in the PHD filter equations indicates how likely the sensor detects an object. Its value depends on the state x, which permits modeling of the occlusion due to other objects present at the scene. To create a model for visibility, we use the radar cross-section (RCS) values reported by the radar for every detected object. As the RCS takes the cross-sectional area of a spherical body with the same reflective capability as the detected object, we model every road participant as a spherical object described by the RCS. If no RCS values are available, a predefined value can be assigned that is appropriate for the considered application, e.g., the cross-sectional area of a typical road vehicle.

**Figure 1:**
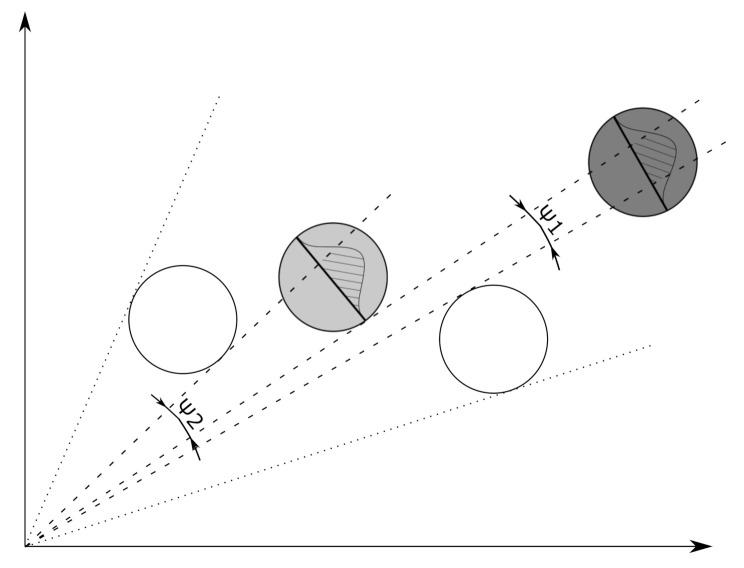
Schematics of the occlusion model used to estimate the detection probabilities of road vehicles. The grey objects are partially covered by the white objects, as seen from the radar’s perspective. The probability of detection is proportional to the hatched area under the curve representing object visibility.

The coefficient $p_{d}\left(x\right)$ represents a probability thus, we regard it as the integral of a PDF ξ that accounts for the visibility. If the whole object lies in the sensor field of view (FOV), and no obstacles are blocking visibility, the PDF integrates to approximate unity. The occlusion model (Figure 1) should set the limits of the integral to compute $p_{d}\left(x\right)$. As we consider a road traffic situation, only planar geometry will be used, hence the angular parametrization of the problem naturally arises.

The probability of detection depends on the state and time-varying, which is manifested through the time-dependent positions of the objects at the scene. $p_{d}$ is expressed as a function of the state and the predicted objects:

(48) $$p_{d,k}\left(x,{\left\{O_{k|k-1,h}^{\left(i\right)}\right\}}_{i=1}^{o_{k-1}}\right)=\int_{\Omega_{k}}\xi \left(\psi \right)d\psi \;,$$

where ψ is the angular parameter. The domain of integration, $\Omega_{k}$ is defined by the sensor FOV and the predicted object set:

(49) $$\Omega \left(x\right)=\mathrm{FOV}-\overset{o_{k-1}}{\underset{i=1}{⋃}}\Psi_{i}\;,$$

where $\Psi_{i}$ is the angular interval covered by object *i*. If a location described by x is closer to the observer than object *i* then $\Psi_{i}$ is empty.

A suitable density function needs to be chosen with the following requirements to model an object’s visibility. It should be bell-shaped with parameters associated with the problem’s geometry and possibly with an analytic cumulative distribution function (CDF) on finite support. The raised cosine distribution with PDF

(50) $$\xi \left(\psi ;m,s\right)=\left\{\begin{array}{cc} \frac{1}{2s}+\frac{1}{2s}\cos\left(\frac{\psi -\mu }{s}\pi \right) & \mathrm{for}-\mu \leq \psi \leq \mu \\ 0 & \mathrm{otherwise} \end{array}\right.$$

is suitable for the problem. The function is centered at μ which is computed from the position coordinates as m=atan2(y,x). The scale parameter *s* is the subtended angle by the diameter of the object which is approximated as the square root of the RCS:

(51) $$s=2\arctan\left(\frac{1}{2}\sqrt{\frac{RCS}{x^{2}+y^{2}}}\right)\;.$$

The CDF of the raised cosine distribution has the form

(52) $$\Xi \left(\psi ;m,s\right)=\frac{1}{2}+\frac{\psi -\mu }{s}+\frac{1}{2\pi }\sin\left(\frac{\psi -\mu }{s}\pi \right)$$

thus the evaluation of the integral (48) is computationally cheap.

## 5. Simulation Results

Various tests were performed to evaluate the performance of the proposed filter. Two abstract scenarios, one with crossing and one with random trajectories, were used to present the filter’s general performance. In these scenarios, the occlusion model was not used, and predefined values were set for the probability of detection. Besides the abstract tests, two situations that model real traffic scenarios were designed. In these setups, the occlusion model was used to estimate the state-dependent probability of detection.

The discrete dynamic system model for a tracked object uses the nearly constant velocity motion model

(53) $$\begin{array}{cc} x_{k+1} & =F_{k}x_{k}+G_{k}w_{k} \end{array}$$

(54) $$\begin{array}{cc} z_{k} & =H_{k}x_{k}+v_{k}\;, \end{array}$$

where the system matrix describes a constant velocity motion with sampling time $T_{s}$:

(55) $$F=I_{2}⊗\left[\begin{array}{cc} 1 & T_{s}\\ 0 & 1 \end{array}\right]$$

and ⊗ denotes the Kronecker product. The state vector has position and velocity components: $x=[x,\dot{x},y,\dot{y}]$. In the following the measurement vector will have the same components as the state vector, thus the measurement matrix is identified: $H_{k}=I_{4}$. The process and measurement noises, $w_{k}$ and $v_{k}$ respectively, are zero mean additive white Gaussian terms with covariances:

(56) $$\begin{array}{cc} \mathrm{Cov}[w_{k}] & =q_{w}^{2}I_{2} \end{array}$$

(57) $$\begin{array}{cc} \mathrm{Cov}[v_{k}] & =I_{2}⊗\left[\begin{array}{cc} \sigma_{1}^{2} & 0\\ 0 & \sigma_{2}^{2} \end{array}\right]\;, \end{array}$$

where $\sigma_{1}$, $\sigma_{2}$ and $q_{w}$ are the noise intensities. The process noise acts through the matrix

(58) $$G=I_{2}⊗\left[\begin{array}{c} \frac{T_{s}^{2}}{2}\\ T_{s} \end{array}\right]\;.$$

### 5.1. Measuring Filter Performance

To compute the multi-object estimation errors, the optimal subpattern assignment (OSPA) metric is used. The OSPA metric was introduced in [38] for the purpose of creating a multi-object estimation error metric. It considers, beside errors in state-space, cardinality differences between the ground truth and the estimated set. Later, the OSPA metric was extended to include labels thus it can measure track estimations errors [39]. The labeled OSPA metric has the form

(59) $$d_{p,c,\alpha }\left(X,Y\right)={\left(\min_{\pi \in \Pi }\frac{1}{n}\sum_{i=1}^{m}d_{c}{\left(x_{i},y_{\pi (i)}\right)}^{p}+\frac{\left(n-m\right)}{n}\cdot c^{p}+\frac{\alpha^{p}}{n}\sum_{i=1}^{m}\left(1-\delta_{\ell \left(x_{i}\right),\ell \left(y_{\hat{\pi }\left(i\right)}\right)}\right)\right)}^{1/p}\;,$$

where p≥1 is the order of the metric, the ground truth set has |X|=m elements and the estimated set cardinality is |Y|=n. Π is the set of permutations of length *m* from elements {1,…,n}. The parameter c>0 is the penalty for a cardinality error and serves as a cutoff value for the base distance:

(60) $$d_{c}\left(x,y\right)=\min\left(c,d\left(x,y\right)\right)\;.$$

The base distance typically, and in this work also, comes from the *p*-norm: $d\left(x,y\right)={∥x-y∥}_{p}$. The value α∈[0,c] is the labeling error parameter and δ is the Kronecker delta. The label associated with state x is denoted by ℓ(x). The optimal permutation $\hat{\pi }$ is given as

(61) $$\hat{\pi }=\arg\min_{\pi }\sum_{i=1}^{n}d_{c}{\left(x_{i},y_{\pi (i)}\right)}^{p}\;.$$

Based on the three terms in (59) the localization, cardinality and labeling errors are:

(62) $$\begin{array}{cc} d_{p,c}^{\mathrm{loc}} & ={\left(\min_{\pi \in \Pi }\frac{1}{n}\sum_{i=1}^{m}d_{c}{\left(x_{i},y_{\pi (i)}\right)}^{p}\right)}^{1/p} \end{array}$$

(63) $$\begin{array}{cc} d_{p,c}^{\mathrm{card}} & =c{\left(\frac{n-m}{n}\right)}^{1/p} \end{array}$$

(64) $$\begin{array}{cc} d_{p,\alpha }^{\mathrm{lab}} & ={\left(\frac{\alpha^{p}}{n}\sum_{i=1}^{m}\left(1-\delta_{\ell \left(x_{i}\right),\ell \left(y_{\hat{\pi }\left(i\right)}\right)}\right)\right)}^{1/p}\;. \end{array}$$

If m>n then *X* and *Y* in (59) should be swapped. For a detailed discussion see [33] (Chapter 6.2).

### 5.2. Abstract Simulations

**Figure 2:**
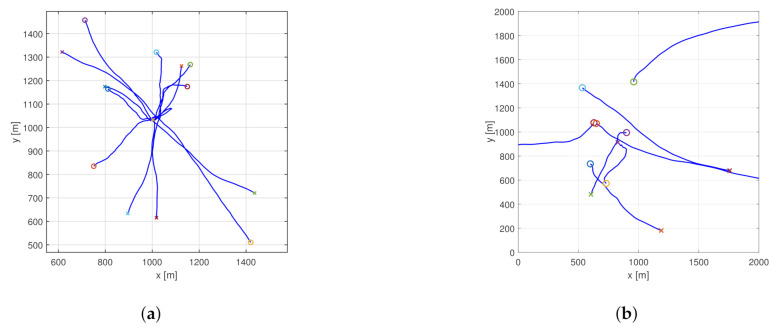
Example scenario ground truth for the crossing trajectories (**a**) and the random trajectories (**b**) simulation. Starting positions are marked with circles, ending positions are indicated by crosses.

In the abstract simulations several objects are moving in the scene (see Figure 2) described by the nearly constant velocity motion model (53). The process noise has intensity $q_{w}=1\;ms^{-1}$. The initial velocity components take values randomly from the interval between −20 and 20 $ms^{-1}$. The standard deviation of the measurement error is $\sigma_{1}=10\;m$ for the position and $\sigma_{2}=1\;ms^{-1}$ for the velocity components.

Although occlusion is not considered, measurement outages are directly inserted into the simulations. For every object, there is a 10 s period when no measurements arrive. These periods are positioned randomly, except for two objects. Periods starting at 10 s, and 25 s were hard-coded for every simulation run. To get reliable performance measures, 200 Monte Carlo simulations were performed, and the error metrics were averaged. The proposed filter’s performance was compared to the PHD filter using the original clustering method without hidden particles. Example scenarios are presented in Figure 2. To evaluate filter performance, a second order OSPA metric was used with a cutoff distance of 100 m and a labeling error of 30 m.

#### 5.2.1. Crossing Trajectories

**Figure 3:**
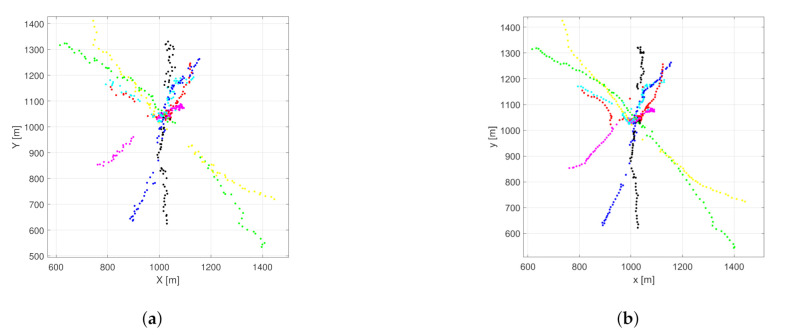
Estimated object positions in the example crossing trajectories scenario using the normal (**a**) and the proposed (**b**) PHD filter.

In the crossing trajectories scenario, seven objects are present. The trajectories are designed in a way that they cross each other around the origin at the same time. Otherwise, the starting and ending positions are random. This setup is challenging regarding object labeling. Figure 3 shows the estimated object positions in an example scenario for the proposed and the basic filter.

**Figure 4:**
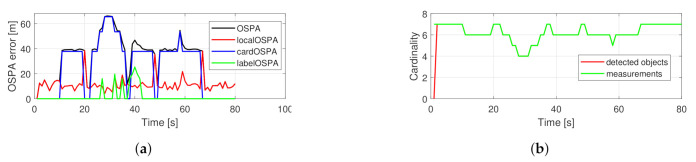
Performance of a single simulation with crossing trajectories using the basic PHD filter. OSPA error (**a**), cardinality statistics (**b**).

**Figure 5:**
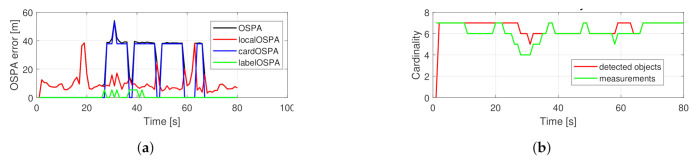
Performance of a single simulation with crossing trajectories using the proposed PHD filter. OSPA error (**a**), cardinality statistics (**b**).

Figure 4 shows the performance of the basic PHD filter in a single run. The OSPA error is dominated by the cardinality component. The number of tracked objects is exactly following the measurement set cardinality. In comparison, the performance of the proposed filter is shown in Figure 5. The localization component in the OSPA error is relatively higher, which is due to the fact that the filter gives estimates about hidden objects, as can be seen around the 20 s. The reduced cardinality and labeling error makes the overall OSPA error less.

**Figure 6:**
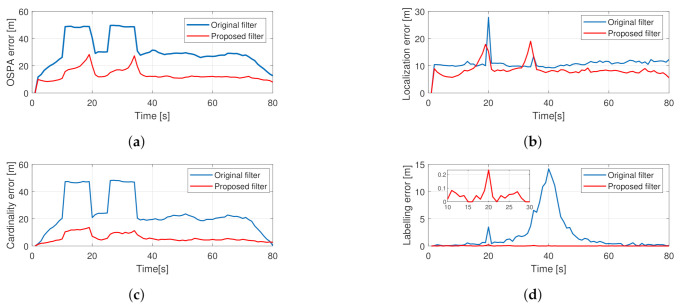
Averaged performance of 200 Monte Carlo simulations from the crossing trajectories scenario using the proposed PHD filter. Total OSPA error (**a**), localization error (**b**), cardinality error (**c**), labeling error (**d**).

The results from the averaged MC runs are shown in Figure 6. The hard-coded outages are observable for the basic filter as two trapezoidal bumps in the cardinality and OSPA error graphs. For the proposed filter, the OSPA error values in the same two regions are less but increase with time. This is due to the increasing localization error while tracking hidden objects and the growing cardinality error, which is the result of the filter dropping tracks as their existence probability falls under the reporting threshold. The labeling error is minimal for the proposed filter, and for the original filter, its maximum value is around 40 s, which is when all trajectories cross each other. A spike that can be seen at the 20th s in the localization error graph for the original filter can be explained by frequent mislabeling of objects, as indicated by a spike in the labeling error graph at the same place.

#### 5.2.2. Random Trajectories

**Figure 7:**
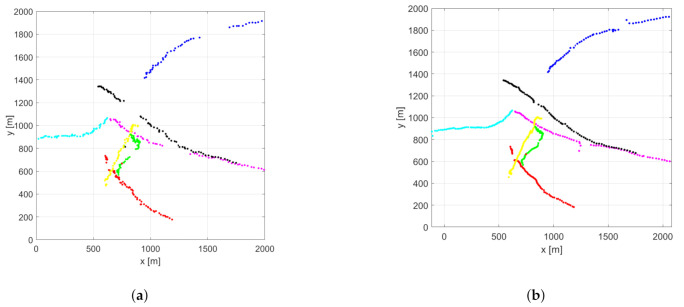
Estimated object positions in the example random trajectories scenario using the normal (**a**) and the proposed (**b**) PHD filter.

In the random trajectories scenario, the objects are moving according to the nearly constant velocity model (53) with random starting positions. Trajectories may or may not cross each other. The measurement outages are designed the same way as described previously. Figure 7 shows the estimated object positions in an example scenario for the proposed and the original filter.

**Figure 8:**
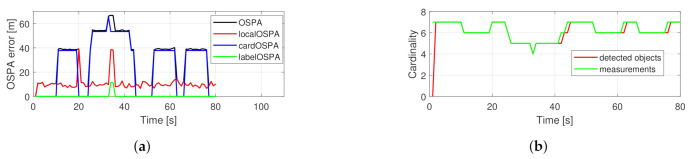
Performance of a single simulation with random trajectories using the basic PHD filter. OSPA error (**a**), cardinality statistics (**b**).

**Figure 9:**
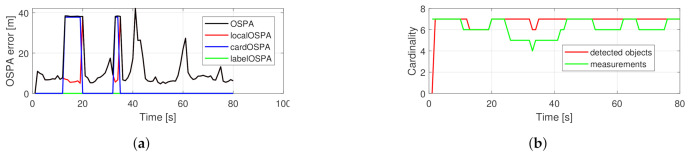
Performance of a single simulation with random trajectories using the proposed PHD filter. OSPA error (**a**), cardinality statistics (**b**).

Figure 8 shows the performance of the original filter for the example scenario. The cardinality component dominates the OSPA error again. The labeling error is smaller because there is less crossing in this setup. In Figure 9 the performance of the proposed filter can be seen. The main components of the OSPA error are the cardinality and localization errors. After the 40th s the localization error has a spike due to subsequent re-detection of two objects.

**Figure 10:**
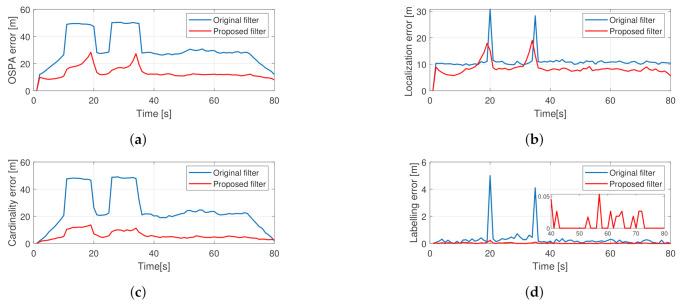
Averaged performance of 200 Monte Carlo simulations from the random trajectories scenario. Total OSPA error (**a**), localization error (**b**), cardinality error (**c**), labeling error (**d**).

The overall filter performances are compared in Figure 10. The advantage of the proposed filter regarding the OSPA error is similar to in the crossing trajectories scenario. Regarding labeling error, the original filter performs reasonably; however, the predefined measurement outages are recognizable. For the proposed filter, labeling errors are minimal.

### 5.3. Road Traffic Simulations

**Figure 11:**
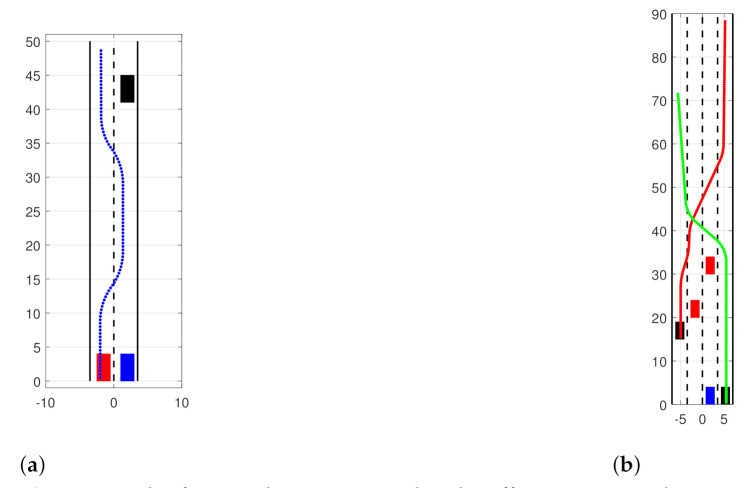
The figures show two simulated traffic scenarios, where a single (**a**) and multiple (**b**) vehicles are occluded for some time by other participants. The ego vehicle is marked with a blue rectangle. Observed vehicles are shown in black while occluding vehicles are red. Colored lines indicate trajectories of moving vehicles. scene1: Object is hidden between 35-64 s scene2: X1 is hidden between 30–43 s and 68-80 s. X2 is hidden between 78–83 Ego vehicle: blue Obstacle: red Observed vehicles filled black Solid line: trajectories.

Two scenarios were designed to evaluate filter performance in simple simulated traffic situations (Figure 11). Rectangles represent the vehicles, and the color indicates whether one is observed (black) or in an occluding object (red). The ego vehicle, drawn as blue, is the observer. Lines represent the trajectories of other vehicles relative to the ego vehicle. The first scenario consists of a two-lane road and three vehicles (Figure 11a). The vehicles in the right lane are moving at the same constant speed. The vehicle in the left lane moves with $5\;ms^{-1}$ speed difference and performs two-lane changing maneuvers, and while moving in the right lane, it occludes the observed vehicle. The simulation’s duration is 100 s, and the observed object is occluded in the time interval [35 s, 64 s].

The second scenario (Figure 11b) consists of a four-lane road segment and four vehicles. The ego vehicles and the two occluding vehicles are moving at the same speed. Two observed vehicles are moving with $5\;ms^{-1}$ relative speed and perform multiple lane-changing maneuvers. The vehicle starting from the right lane is occluded in the time interval of [30 s, 43 s] and [68 s, 80 s]. The other observed vehicle is occluded in the time interval [78 s, 83 s]. To evaluate filter performance, a second order OSPA metric was used with a cutoff distance of 30 m and labeling error of 10 m; however, no labeling error occurred.

**Figure 12:**
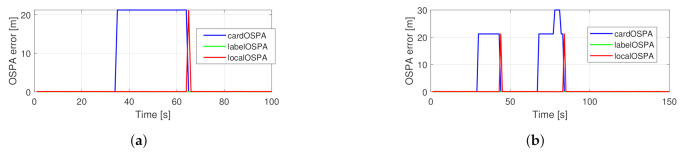
Performance of the basic filter in the simulated traffic scenarios with one (**a**) and two (**b**) observed vehicles.

**Figure 13:**
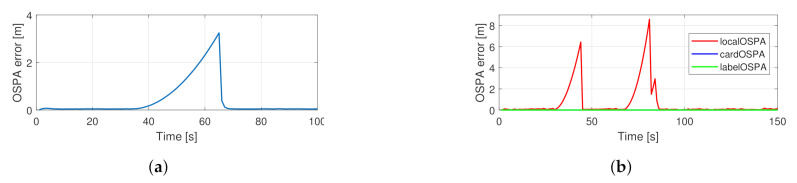
Performance of the proposed filter in the simulated traffic scenarios with one (**a**) and two (**b**) observed vehicles.

The original filter’s performance in the simulated traffic situations can be seen in Figure 12. As expected, during occlusion, the OSPA error is dominated by the cardinality component, and after re-detection, the localization error component is significant. The performance of the proposed filter is shown in Figure 13. In this case, no cardinality error occurs, and the localization error increases gradually as the estimation becomes more uncertain during occlusion. The actual scaling of the cardinality error depends on the cutoff distance, hence the information gain manifested through the correct cardinality estimates should be tuned via this parameter.

## 6. Highway Measurements

The effectiveness of the proposed filter is validated using highway radar measurements acquired in traffic. The road segment used is the Hungarian M1 motorway. The vehicle is equipped with a Mobileye 630 camera and an automotive radar (Continental ARS408-21). The camera is used to give lane information and to provide approximate ground truth for visual checking. The filter is only supplied with the radar measurements. The radar communicates via CAN bus and provides information about the detected objects in the environment. Every object is assigned an ID, an integer number which remains constant while the object lives continuously. When an object disappears and then reappears, the previous ID may or may not be assigned again. It depends on whether the radar identifies that object the same as seen previously or considers it a new one. The old ID may be assigned to another object that appeared or moved near the location where one disappeared. This radar behavior is directly addressed in the proposed filter; thus, we can get continuous object trajectories. The measurements are logged using a Vector CAN device and processed offline in Matlab. The filter is equipped with the lateral and longitudinal object position and velocity coordinates, the IDs, and RCS values. The filtering timestep is 0.1 s.

Due to the high refresh rate, the radar detects and reports an object multiple times during the filtering timestep. Instead of using a non-standard measurement model that captures this peculiarity, we process the measurements online to handle the numerous detections. We collect the measurements that arrived during the filter timestep and form unique clusters. We use the fact that vehicles cannot overlap in the position subspace of the state-space. The algorithm is summarized in Algorithm 1.

**Figure 14:**
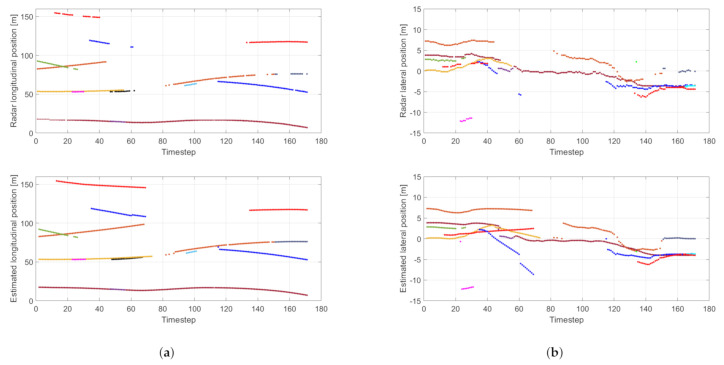
Lateral (**a**) and longitudinal (**b**) trajectory components of the detected objects. Top figures show raw radar measurements, bottom figures show estimated values.

**Figure 15:**
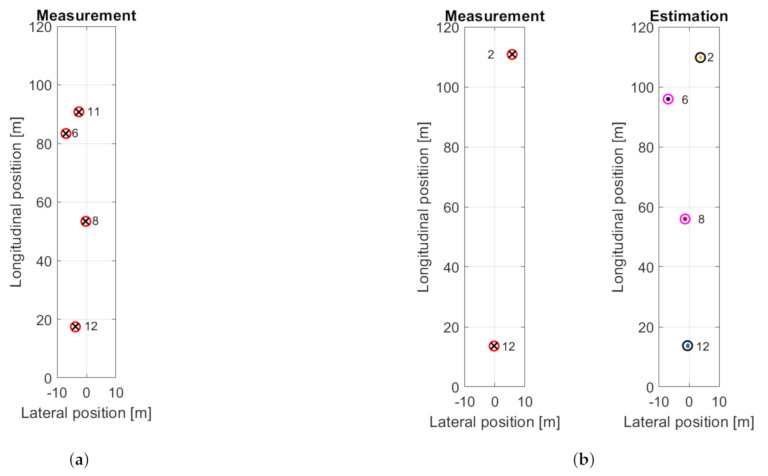
Vehicle positions at the beginning (**a**) and during lane-changing maneuver (**b**), when vehicle 12 occludes vehicle 6 and 8.

The traffic scenario is the following. At the beginning the ego vehicle is in the left lane of the three-lane motorway. There are four other observable vehicles (see Figure 14 and Figure 15a). The one in the middle lane, about 20 m ahead (bottom trajectory in the longitudinal plots, with ID 12) remains in that lane and moves with constant speed during the measurement. The ego vehicle performs a double lane-changing maneuver and arrives to right lane while the vehicle in the middle occludes other traffic participants (Figure 15b). The vehicle in the right lane, about 55 m ahead at the beginning, labeled with ID 8, also performs a double lane-changing maneuver. The vehicle in the left lane (ID 6) moves with constant speed and remains in that lane.

Figure 15b belongs to timestep 60 where there are two radar measurements and six estimated objects. Please note that two objects are out of the plot range: the red trajectory at 150 m longitudinal position and the black trajectory at 20 m lateral position in Figure 14.

Figure 14 shows the unprocessed (top figures), and the estimated (bottom figures) object positions in a sequence. In several cases, the filter gives continuous trajectories for temporarily occluded objects. In these cases, a jump can be observed when the object reappears because of correcting the state estimates based purely on the motion model. This can be observed for the blue trajectory at timestep 60. The filter drops the ceased trajectories with a time delay, which is a natural consequence of the proposed method: memory is inserted into the PHD filter that manifests as if inertia is associated with the hypotheses of object tracks. Track initialization is, however, not delayed by this inertial effect.

Considering the maneuvers of the ego vehicle and inserting its motion into the dynamic model would help the filter to get better state estimates during outages as trajectory shifts would be anticipated.

## 7. Conclusions

The presented work has three contributions that aim to address the limitations of the classical PHD filter. Using the standard measurement model, we proposed a method to handle multiple detections of the same object in a single time frame. By adopting a model to estimate the state-dependent detection probability, we could reduce the effect of missed detection on the cardinality estimate. Regarding track management and reporting of detected objects, the proposed method introduces inertia, which the classical PHD filter lacks, to gain additional cardinality estimate stability. The presented algorithm’s performance is evaluated in synthetic tests using simulations and logged sensor data originating from real-world traffic situations. Besides the state-dependent probability of detection, the filter could benefit from the introduction of state-dependent survival probabilities that would allow the modeling of objects exiting the scene or discard objects in irrelevant regions. Modeling the ego vehicle motion is another possible extension as it would make tracking more effective during a lane-changing maneuver.

## Data Availability

Measurement data is available at the authors.

## Abbreviations

The following abbreviations are used in this manuscript:

PDF	probability density function
CDF	cumulative distribution function
GM	Gaussian mixture
PF	particle filter
SMC	sequential Monte Carlo
FISST	finite set statistics
RFS	random finite set
PHD	probability hypothesis density
JPDAF	joint probabilistic data association filter
MHT	multiple hypothesis tracking
OSPA	optimal subpattern assignment
FOV	field of view
